# Soluble CD40 ligand directly alters glomerular permeability and may act as a circulating permeability factor in FSGS

**DOI:** 10.1371/journal.pone.0188045

**Published:** 2017-11-20

**Authors:** Sophie Doublier, Cristina Zennaro, Luca Musante, Tiziana Spatola, Giovanni Candiano, Maurizio Bruschi, Luca Besso, Massimo Cedrino, Michele Carraro, Gian Marco Ghiggeri, Giovanni Camussi, Enrico Lupia

**Affiliations:** 1 Department of Oncology, University of Turin, Turin, Italy; 2 Department of Medical Sciences, University of Turin, Turin, Italy; 3 Department of Medical, Surgery and Health Sciences, University of Trieste, Trieste, Italy; 4 Nephrology, Dialysis, Transplantation and Laboratory on Pathophysiology of Uremia, G. Gaslini Children Hospital, Genoa, Italy; University of Houston, UNITED STATES

## Abstract

CD40/CD40 ligand (CD40L) dyad, a co-stimulatory bi-molecular complex involved in the adaptive immune response, has also potent pro-inflammatory actions in haematopoietic and non-haematopoietic cells. We describe here a novel role for soluble CD40L (sCD40L) as modifier of glomerular permselectivity directly acting on glomerular epithelial cells (GECs). We found that stimulation of CD40, constitutively expressed on GEC cell membrane, by the sCD40L rapidly induced redistribution and loss of nephrin in GECs, and increased albumin permeability in isolated rat glomeruli. Pre-treatment with inhibitors of CD40-CD40L interaction completely prevented these effects. Furthermore, *in vivo* injection of sCD40L induced a significant reduction of nephrin and podocin expression in mouse glomeruli, although no significant increase of urine protein/creatinine ratio was observed after *in vivo* injection. The same effects were induced by plasma factors partially purified from post-transplant plasma exchange eluates of patients with focal segmental glomerulosclerosis (FSGS), and were blocked by CD40-CD40L inhibitors. Moreover, 17 and 34 kDa sCD40L isoforms were detected in the same plasmapheresis eluates by Western blotting. Finally, the levels of sCD40Lwere significantly increased in serum of children both with steroid-sensitive and steroid-resistant nephrotic syndrome (NS), and in adult patients with biopsy-proven FSGS, compared to healthy subjects, but neither in children with congenital NS nor in patients with membranous nephropathy.

Our results demonstrate that sCD40L directly modifies nephrin and podocin distribution in GECs. Moreover, they suggest that sCD40L contained in plasmapheresis eluates from FSGS patients with post-transplant recurrence may contribute, presumably cooperating with other mediators, to FSGS pathogenesis by modulating glomerular permeability.

## Introduction

The CD40/CD40 ligand (CD40L) dyad, a bi-molecular component of the tumor necrosis factor (TNF) gene superfamily, plays a critical role in the adaptive immune response [1–4]. Both molecules have widespread distribution: CD40L is preferentially expressed on activated CD4^+^ T lymphocytes and platelets, and CD40 on B lymphocytes, monocytes/macrophages, and dendritic cells. In addition, both CD40 and CD40L are expressed by several non-haematopoietic cells, such as endothelial and smooth muscle cells (SMCs) [1–4]. Moreover, a truncated soluble form of CD40L (sCD40L), derived from proteolytic cleavage of CD40L, can be detected in the circulation [1–4].

The ligation of CD40 by sCD40L on endothelial cells, SMCs, monocytes, and dendritic cells results in induction of adhesion molecules and production of pro-inflammatory cytokines, chemokines, matrix metalloproteinases and tissue factor [1–4], with relevant implications in the pathogenesis of cardiovascular, immunological and neoplastic diseases [1–4].

In renal pathophysiology, blockade of CD40-CD40L interaction has a protective effect in allograft rejection [5] and in several models of experimental glomerulonephritis, with a mechanism mediated by T-cell immunity [6–9]. CD40 is expressed by mesangial and tubular cells, and its stimulation activates a variety of pro-inflammatory responses [10–13]. Although CD40 expression by glomerular epithelial cells (GECs)/podocytes has been recently shown by several research groups [9, 14–17], little is known on its physiologic function in this cell-type, and on its involvement in the pathogenesis of podocytopathies, a distinct group of diseases characterized by a functional modification of renal permselectivity, with proteinuria and nephrotic syndrome as clinical hallmarks, and minimal or focal segmental glomerulosclerosis lesions (MCN or FSGS, respectively) as their pathology backgrounds. Post-transplant recurrence of proteinuria occurs in almost 50% of FSGS patients receiving a renal graft with a kinetic of proteinuria (frequently occurring within few minutes from transplant) that recalls the existence of a circulating plasmatic permeability factor. This is an area of intense research that has produced so far inconsistent results [18–29].

On the basis of preliminary proteomic approach to plasmapheresis eluates from patients with abrupt recurrence of proteinuria after a renal graft, and of the recent description of agonistic anti-CD40 antibodies in FSGS patients [14], we hypothesized that sCD40L could act as a permeability trigger of recurrence. Therefore, we studied the effect of sCD40L on nephrin expression and cytoskeletal organization in cultured podocytes, on the permselectivity of isolated rat glomeruli; on nephrin and podocin glomerular expression and proteinuria induction after *in vivo* injection in mice. In addition, we investigated whether the inhibition of CD40-CD40L interaction prevents the effects induced in cultured podocytes and in isolated glomeruli by plasma fractions purified from plasmapheresis eluates obtained from patients with post-tranplant recurrence of FSGS, and we measured the circulating levels of sCD40L in patients affected by FSGS compared to healthy subjects and to patients with membranous nephropathy.

## Materials and methods

### Reagents

Human recombinant soluble CD40L (hr-sCD40L) trimeric protein plus enhancer (cross-linking Ab), mouse recombinant soluble CD40L (mr-sCD40L) set, recombinant CD40-murine Ig (muIg) fusion protein, consisting of the extracellular domain of human CD40 fused to mouse IgG2a, recombinant human CD40L-muCD8 fusion protein, constituted by murine CD8 fused to human CD40L, and anti-human CD40L mAbs (MK13A4 and 24–31) were obtained from Alexis Biochemicals (San Diego, CA).

Rabbit anti-CD40 polyclonal antibody (H-120) used for western blot, and mouse anti-CD40 mAb used for indirect immunofluorescence and flow cytometry experiments were from Santa Cruz Biotechnology (Santa Cruz, CA) and from BD Biosciences (Bedford, MA), respectively.

Anti-CD40 rabbit polyclonal antibody used for indirect immunofluorescence on frozen mouse kidney sections was from Abcam, Cambridge, UK (ab58391).

### Patients

For the experiments on plasma “Permeability Factor” (PF), we used six plasmapheresis eluates of FSGS patients who presented active post-transplant recurrence (see Table 1 for their clinical characteristics). Plasmapheresis eluates used in this portion of the study was obtained within 3 to 6 days from proteinuria recurrence. All patients were treated with *i*.*v*. steroids and calcineurin inhibitors at the time of apheresis. The methods used to prepare a partially purified PF from plasmapheresis eluates, which was employed in the following *in vitro* experiments, are detailed in a separate paragraph.

**Table 1:** Baseline characteristics of patients with FSGS who received a renal transplant and presented recurrence of proteinuria. All patients were treated with plasmapheresis and had their plasmapheresis eluates processed for partial purification of Permeability Factor (PF). P_alb_ activity reported in the Table is referred to that measured in the partially purified fraction prepared from plasmapheresis eluates following a procedure based on protein A Sepharose and differential precipitation in ammonium sulphate (see Methods for details).

Patient N.	Sex	Age at onset (yrs)	Histological pattern	Age at Tx (yrs)	Recurrence (months after Tx)	Therapy	P_alb_	*Ur Prot* *(gr/day)*	*S Creat* *(mg%)*	*Outcome*
**1**	F	14	FSGS	19	6	S; FK; MMF	0.85	3	0.9	Remission
**2**	M	5	FSGS	12	4	S; FK; MMF	0.9	2	1.5	CRF
**3**	M	10	FSGS	25	1	S; FK	0.8	5	2	Remission
**4**	M	2	FSGS	6	5	S; FK; MMF	0.75	4	0.5	Remission
**5**	M	4	FSGS	11	6	S; FK	0.8	4	0.6	Remission
**6**	M	2	FSGS	7	0.5	S; FK	0.7	8	0.6	CRF

**Legends,** Tx, transplantation; S, steroids; FK, Tacrolimus; MMF, Mycophenolate Mofetil.

Circulating levels of serum sCD40L were measured in several cohorts of children and adult patients with NS subdivided according to the sensitivity to steroids, defined as normalization of proteinuria (<150 mg/24h, Prot/Creat < .2) after 30 days of therapy with 60 mg/m^2^prednisone. In those cases showing steroid resistance, a second line drug was started, generally calcineurin inhibitors (Cyclosporin 4mg/Kg/d, or Tacrolimus 0.1 mg/Kg/d), and, in case of persisting proteinuria, a renal biopsy was performed.

Children who developed NS under the 1^st^ year of age were defined as congenital nephrotic syndrome (cNS). All of them underwent a bioptic approach, and were studied with molecular tests for NPHS1, NPHS2, WT1, PLCE1, ACTN4, TRPC6, and CD2AP.

All adults with NS had a renal biopsy at the onset of proteinuria, and then they were treated with steroids in association with calcineurin inhibitors.

Overall, 96 patients were studied: 23 children with steroid-dependent NS (SDNS); 15 children with steroid-resistant NS (SRNS), all having a histology diagnosis of FSGS; 10 adults (aged >40 years) with SRNS, all with a renal biopsy proving FSGS; 8 patients with congenital NS (age at onset <1 year); 40 adults with idiopathic Membranous Nephropathy (Table 2). All patients had an eGFR greater than 60 ml/min at the time of sampling.

**Table 2:** IDIOPATHIC NEPHROTIC SYNDROME. Baseline characteristics of patients affected by idiopathic nephrotic syndrome who underwent evaluation of serum levels of sCD40L.

	SDNS	SRNS		Congenital NS	iMN
Cases	<18 yrs N = 23	<18 yrs N = 15	>18yrs N = 10	N = 8	N = 40
Age—yrs (range)	9.8 (4–23)	9.3 (3.8–22)	63 (41–78)	9 (2–18)	42 (26–64)
Age at onset–yrs (range)	2.9 (0.2–7.1)	3.6 (1.5–14)		<1	36 (26–52)
**Male sex** (%)	17 (74%)	7 (47%)	5 (50%)	3 (38%)	24 (60%)
**Renal Histology** (%)					
Not performed*	13 (57%)	0	0	0	0
*FSGS*	0	15 (100%)	10 (100%)	8 (100%)	0
*IgM*	4	0	0	0	0
*MCD*	4	0	0	0	0
*MN*	-	-	0	0	40
Steroid sensitivity	23 (100%)	0	0	0	-
CTX therapy (%)	2 (9%)	2 (13%)	0	0	0
FK506 or Cyclosporine (%)	15 (65%)	15 (100%)	1 (10%)	0	4 (10%)
ARB or ACEI (%)	0	12 (80%)	10 (100%)	8 (100%)	40 (100%)
Urinary protein >1g/m^2^/day	18 (78%)	15 (100%)	10 (100%)	8 (100%)	40 (100%)

* Renal biopsy was not performed in patients who had presented at least an episode of drug responsiveness in the past.

Median (range) was reported for quantitative variables, and absolute (relative) frequencies for qualitative variables.

***Legend*:** FSGS: Focal and Segmental Glomerular Sclerosis; MCD: Minimal Change Disease; MN: membranous nephropathy; CTX therapy: previous use of cytotoxic agents (Endoxan^R^, Leukeran^R^); FK506: tacrolimus; ARB/ACEI: angiotensin receptor blockers or converting enzyme inhibitors.

All patients were in a pre-transplant phase and presented variable degree of proteinuria.

As controls, we also studied 11 healthy volunteers, receiving no medications and with haematological indices, and liver and kidney function tests (including proteinuria) within normal ranges.

The study was conducted according to the Helsinki Declaration, and approved by the Institutional Ethical Committee of G. Gaslini Children Hospital, Genoa (EudraCT: 2008-004486-26). All patients or, for minors/children, next of kin, caretakers, or guardians signed a written informed consent form for the participation in the study.

### Culture of glomerular epithelial cells (GECs)/podocytes

Primary cultures of human glomerular epithelial cells (GECs)/podocytes were established and characterized as previously described [30]. Established lines of differentiated GECs were obtained by infection of primary cultures with a hybrid Adeno5/SV40 virus as previously described and characterized [30, 31].

Primary rat and mouse podocytes were established as previously described [32].

### Preparation of partially purified Permeability Factor (PF) from plasmapheresis eluates of FSGS patients

Partially purified PF was prepared from plasmapheresis eluates following a procedure based on protein A Sepharose and differential precipitation in ammonium sulphate [33]. Plasmapheresis eluates were first passed through a 20x5 cm column of Protein A Sepharose (Amersham, Aylesbury, UK) and, after washings with glycine buffer pH 2.5, PF were eluted with 6 M guanidine pH 9. The material bound to Protein A was precipitated with 80% ammonium sulphate and the supernatant was utilized for *in vitro* studies. At every step of the procedure, separate chromatographic fractions were tested with a bioassay on isolated glomeruli (see below) for the presence of permeability activity (P_alb_), and only those fractions showing P_alb_> 0.5 were further processed. Protein concentration was determined with the Coomassie Dye binding assay described by Bradford [34]. Overall, P_alb_ of the final product was higher than in whole plasma by a factor of 1,000 [33].

### Flow cytometry

For flow cytometry analysis, GECs were washed in cold PBS and detached using nonenzymatic method. After incubation with mouse anti-CD40 mAb (1:100), followed by FITC-conjugated rabbit anti-mouse secondary antibody, the cells were analysed for relative fluorescence intensity on a Becton Dickinson FACStar-plus instrument using appropriate gating excluding dead cell population. Data analysis used a FACS plot program (WinMDI by Joseph Trotter).

### Immunofluorescence on cultured GECs

Indirect immunofluorescence on cultured GECs was performed as previously described [35].

### RT-PCR

RT-PCR was performed using standard procedure. Briefly, total RNA was extracted using TRI reagent (Sigma Chemical Co, St. Louis, MO), and the RNA pellet dissolved in 10 μl of diethyl pyrocarbonate water and stored at -70°C. 1 μg of total RNA was reverse-transcribed using a First Strand Synthesis Kit (Boehringer Mannheim, Indianapolis, IN). Sequence-specific oligonucleotide primers were designed (human CD40: forward 5′ CCT CGC CAT GGT TCG TCT GCC, reverse 5′ AGC CAG GAA GAT CGT CGG GA [36]; rat CD40: forward 5′GTGTGTTACGTGCAGTGACAA, reverse 5′ATCCTCACAGCTTGT CCA [37]; mouse CD40: forward 5′TGGTCATTCCTGTCGTGATG, reverse 5′GGCTCTGTCTTGGCTCATCT [38]). Times and temperatures for denaturation, annealing and extension were 1 minute 94°C, 1 minute 55°Cor 57°C or 60°C respectively for human, rat, or mouse CD40, and 2 minutes 72°C. Amplification products were visualised by ethidium bromide staining after agarose gel electrophoresis.

### Western blotting

Nephrin and CD40 expression in GECs was also evaluated by Western blot analysis [35] using anti-nephrin mAb (at 2.5 μg/ml) or rabbit anti-CD40 polyclonal antibody (1:200), respectively, as previously described [39].

For the detection of sCD40L in PF partially purified from plasmapheresis eluates from patients with FSGS, different PF preparations (5 μg), each derived from a single patient, were subjected to 10% SDS-PAGE under reducing conditions, and, after transfer to nitrocellulose membranes, blotted with anti-human CD40L mAb (24–31; 1:1000).

### Measurement of albumin permeability activity (P_alb_) in isolated glomeruli

To measure the permeability activity to albumin induced by hr-sCD40L or PF, we used the method described by Savin et al. [18, 40], with minor variations [19, 33]. Briefly, isolated rat glomeruli were incubated at 37°C in 200 μl of medium containing hr-sCD40L (100 ng/ml; + 1 μg/ml of enhancer) or PF (500 ng/ml). The medium also contained 5 g/dl bovine serum albumin (BSA; Sigma Chemical Co) as an oncotic agent. Albumin permeability is a variable with a minimum of 0.00 in normal glomeruli and a maximum of 1.00 after injury to the permeability barrier. P_alb_ values > 0.5 are considered significantly elevated.

This portion of the study was carried out at the University of Trieste after approval of the experimental protocol by a Committee of Italian Health Ministry (Prot. N. 1120 –August, 7, 2012), and in compliance with Italian regulations (D.L.vo 116/92).

### Experimental in vitro conditions

For immunofluorescence (IF) studies, GECs were plated in eight-well Permanox slide at a density of 50,000 cells per well in DMEM 10% FCS.

In the experiments aimed to study nephrin expression, GECs were incubated with hr-sCD40L trimeric protein and enhancer (100 ng/ml; + 1 μg/ml of the cross-linking antibody) or PF (500 ng/ml) for variable times, prior to fixation and staining with mAb specific for the extracellular domain of nephrin.

To evaluate actin microfilament, GECs were fixed after stimulation, permeabilized by incubation for 5 min at 4°C in HEPES-Triton X-100 buffer, and stained for 30 min at 37°C with FITC-phalloidin (2 μg/ml).

In order to block the biological effects of sCD40L, hr-sCD40L or PF were pre-treated for 10 minutes with CD40-muIg fusion protein (20 ng/ml) or with an anti-human CD40L blocking mAb (5 μg/ml), or either GECs or isolated rat glomeruli were pre-treated for 30 minutes with recombinant human CD40L-muCD8 fusion protein (50 ng/ml).

### In vivo injection of recombinant sCD40L or PF from plasmapheresis eluates of FSGS patients

Mouse recombinant sCD40L (200 ng) or PF (approximately 15 μg) obtained from plasmapheresis eluates from FSGS patients, both diluted in a final volume of 100 μl PBS containing 5% bovine serum albumin (BSA), was injected into the tail vein in female C57BL/6 or SCID mice, respectively. As negative control, we used heat-inactivated sCD40L or heat-inactivated PF, as appropriate, or PBS containing 5% BSA. Urine was collected before and after sCD40L, PF or BSA injection for protein and creatinine analysis. Twenty-four hours after injection, mice were killed by using deep inhalation anesthesia (isoflurane) followed by an overdose of barbiturate (thiopental), and kidneys snap-frozen for immunofluorescence studies.

This portion of the study was carried out at the University of Turin in strict accordance with the recommendations in the Guide for the Care and Use of Laboratory Animals of the National Institutes of Health, and guidelines and Italian government regulations. The protocol was approved by the Institutional Review Committee for Animal Care and Use at the University of Turin (Protocol Number: 0008619-P).

### Immunofluorescence studies on kidney sections

4-**μ**m-thick cryostat sections from mouse kidney (6 per group) were incubated overnight at 4°C with an anti-CD40 rabbit polyclonal antibody (ab58391; Abcam, Cambridge, UK), or an anti-nephrin antibody (GP-N1; ProgenBiotechnic, Heidelberg, Germany; 1:100), or an anti-podocin antibody (H-130; Santa Cruz Biotechnology; 1:100), followed by the appropriate secondary antibody (Alexa Fluor 488 anti-guinea pig or anti-rabbit; Molecular Probes, Leiden, the Netherlands). Hoechst 33258 dye (Sigma) was added for nuclear staining. The number of glomeruli available on each section ranged between 5 and 10.

Confocal microscopy analysis was performed using a Zeiss LSM 5 Pascal Model Confocal Microscope (Carl Zeiss International, Jena, Germany).

Nephrin and podocin expression were analysed semi-quantitatively as previously described [35].

### Measurement of serum sCD40L levels

sCD40L was measured in duplicate using an ELISA assay (R&D Systems, Minneapolis, Minnesota), following the manufacturer’s instructions.

### Data analysis

Data represent means ± standard error. Statistical analyses were performed with GraphPad Prism 4.00 for Windows (GraphPad Software, La Jolla, CA, USA) using the Student’s t test or one-way analysis of variance (ANOVA) in combination with Dunn’s multiple comparison test, as appropriate.

Correlation between variables was assessed by Pearson correlation including data from all groups. A P value of < 0.05 was considered significant.

## Results

### Expression of CD40 in glomerular epithelial cells (GECs)

**Fig 1:**
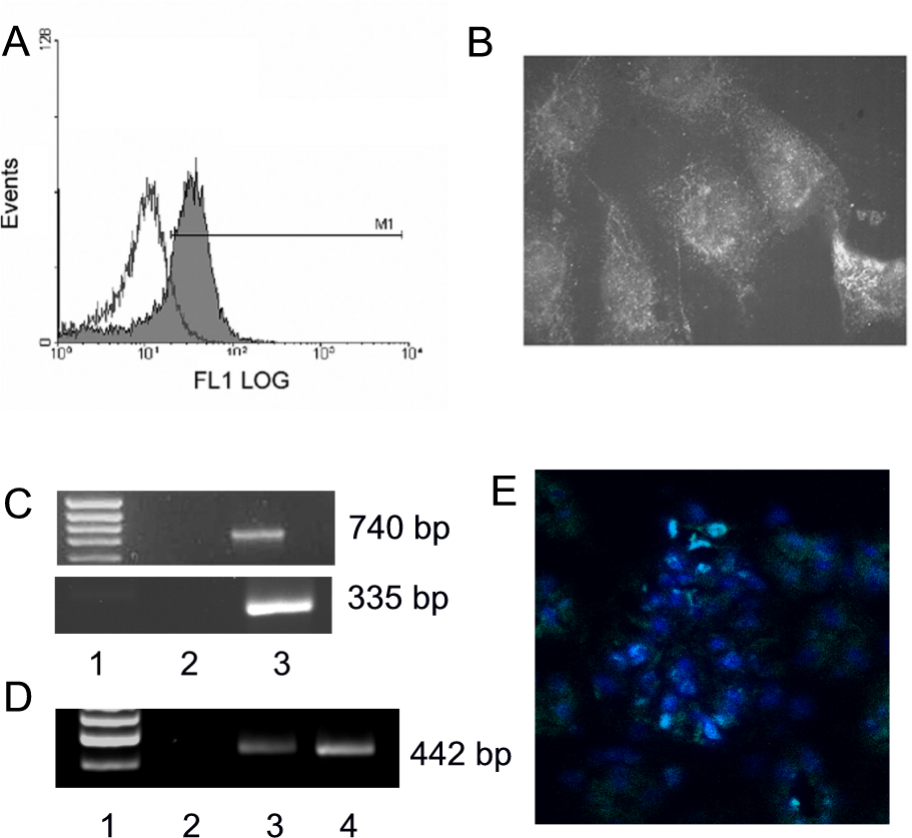
Expression of CD40 in glomeruli and cultured GECs. Panel A. Detection of CD40 by flow cytometry in cultured human GECs. Panel B. Indirect immunofluorescence staining for CD40 in cultured human GECs (x400). Panel C. Representative PCR gels for CD40 expression in cultured human (upper panel) and mouse (lower panel) GECs. Lane 1: 100 bp molecular weight markers; Lane 2: PCR for CD40, no cDNA control; Lane 3: PCR for CD40. Panel D. Representative PCR gels for CD40 expression in primary cultures of rat GECs and rat glomeruli. Lane 1: 100 bp molecular weight markers; Lane 2: PCR for CD40, no cDNA control; Lane 3: PCR for CD40 on primary cultures of rat GECs. Lane 4: PCR for CD40 on rat glomeruli. Panel E. Indirect immunofluorescence staining for CD40 in frozen mouse kidney sections (x63). Images are representative of at least 3 separate experiments with similar results.

We demonstrated mRNA and protein expression of CD40 in both primary cultures and in established lines of differentiated human GECs by multiple techniques, including flow cytometry (Fig 1A), indirect immunofluorescence (Fig 1B) and RT-PCR (Fig 1C, upper image). In addition, we have shown by RT-PCR that primary cultures of rat and mouse GECs and rat glomeruli also express CD40 (Fig 1, panel C, lower image, and panel D). Finally, podocyte CD40 expression was shown in mouse frozen kidney sections by indirect immunofluorescence (Fig 1E).

### Effect of sCD40L on nephrin expression and cytoskeleton organization in cultured GECs

**Fig 2:**
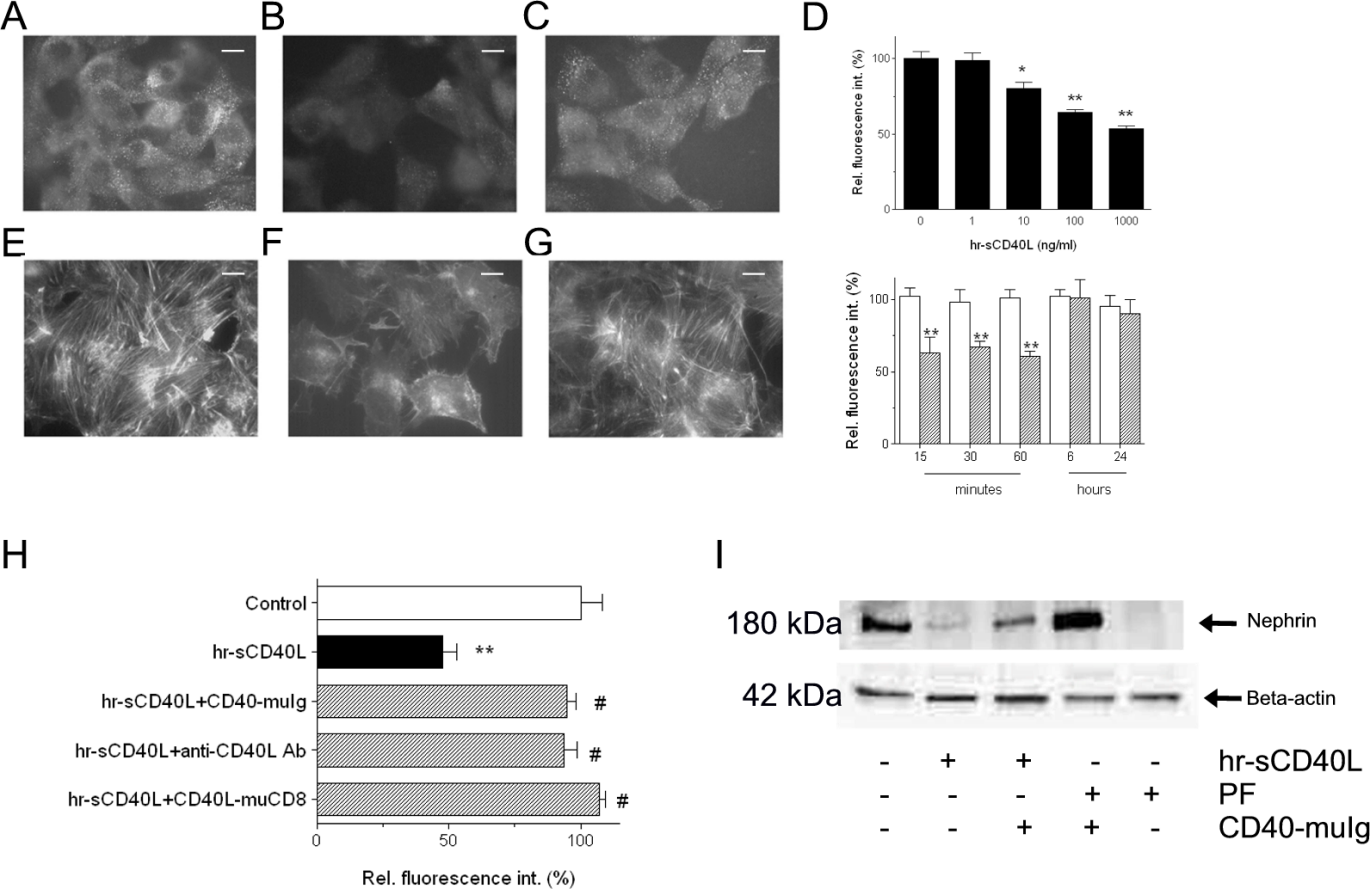
Effect of sCD40L on nephrin expression and cytoskeleton organization in cultured GECs. (A-C) Micrographs representative of immunofluorescence staining for nephrin in non-permeabilized GECs: (A) GECs incubated with vehicle alone for 30 min; (B) GECs incubated with hr-sCD40L (100 ng/ml + 1 μg/ml enhancer) for 30 min; (C) Effect of pretreatment with an inhibitor of CD40-CD40L interaction, CD40-muIg fusion protein (20 ng/ml), on loss of nephrin induced by hr-sCD40L. Original magnification: ×400 (F-H). Bars = 10 μm. Images are representative of at least 5 separate experiments with similar results. (D) Semiquantitative analysis of nephrin expression as detected by immunofluorescence staining in GECs incubated with various concentrations of hr-sCD40L for 30 min (upper graph), and of the time-course effect of incubation of GECs with hr-sCD40L (100 ng/ml + 1 μg/ml enhancer; dashed bars) or vehicle alone (open bars) on nephrin expression, as detected by immunofluorescence staining (lower graph). Values are derived from 5 or more experiments for each experimental condition and expressed as percent variations from baseline value. (E-G) Micrographs representative of fluorescein isothiocyanate phalloidin staining of actin microfilaments in permeabilized GECs. (E) GECs incubated with vehicle alone for 30 min; (F) GECs incubated with hr-sCD40L (100 ng/ml + 1 μg/ml enhancer) for 30 min; (G) Effect of pretreatment with an inhibitor of CD40-CD40L interaction, CD40-muIg (20 ng/ml), on reorganization of cytoskeleton induced by hr-sCD40L. Original magnification: ×400 (D-I). Bars = 10 μm. Images are representative of at least 5 separate experiments with similar results. (H) Semiquantitative analysis of nephrin expression as detected by immunofluorescence staining in GECs incubated with hr-sCD40L (100 ng/ml + 1 μg/ml enhancer) for 30 min in the absence (black bar) or presence of different inhibitors of CD40-CD40L interaction (dashed bars). Using CD40-muIg fusion protein (20 ng/ml) and the neutralizing antibody against CD40L (5 μg/ml), hr-sCD40L was pre-treated for 10 minutes prior to GEC stimulation, whereas the CD40L-muCD8 fusion protein (50 ng/ml) was added to cultured GECs 30 minutes before adding hr-sCD40L (see Methods for details). Values are derived from 5 or more experiments for each experimental condition and expressed as percent variations from baseline value. (I) Immunoblot of a representative experiment on the effect of hr-sCD40L and PF on nephrin expression as detected by Western blot analysis. GEC lysates were immunoblotted with antibodies anti-nephrin or Beta-actin after incubation with hr-sCD40L (100 ng/ml + 1 μg/ml enhancer) or PF (500 ng/ml), in the presence or absence of CD40-muIg fusion protein (20 ng/ml) to inhibit CD40-CD40L interaction. Blots are representative of three independent experiments with similar results.

We have then evaluated, in cultured podocytes, the effect of CD40 stimulation by human recombinant sCD40L on nephrin expression and cytoskeleton organization, phenomena that had been observed to be induced by other well-known proteinuric agents [35, 39]. sCD40L induced a significant reduction of nephrin expression on podocyte cell membrane (Fig 2, panels B and D), evident after only 15 minutes (Fig 2D, lower graph). This decrease of nephrin expression was transient, as nephrin was re-expressed after 6 and 24 hours (Fig 2D, lower graph). The pre-treatment of sCD40L with an inhibitory CD40-muIg fusion protein or with a neutralizing antibody against CD40L, or of GECs with a CD40L-muCD8 fusion protein prevented the disappearance of nephrin from podocyte surface (Fig 2, panels C and H).

In parallel to nephrin re-distribution and loss from GEC membrane, sCD40L induced a deep cytoskeleton reorganization, revealed by loss in stress fibers, cortical accumulation of F-actin and cell retraction (Fig 2F), which was blunted by inhibiting CD40-CD40L interaction (Fig 2G). Finally, nephrin was barely detectable by Western blot in cell lysates from sCD40L-stimulated GECs, whereas it appeared preserved in cell lysates obtained from GECs that were pre-treated with inhibitors ofCD40-CD40L interaction (Fig 2I).

### Effect of sCD40L on glomerular permselectivity in isolated rat glomeruli

**Fig 3:**
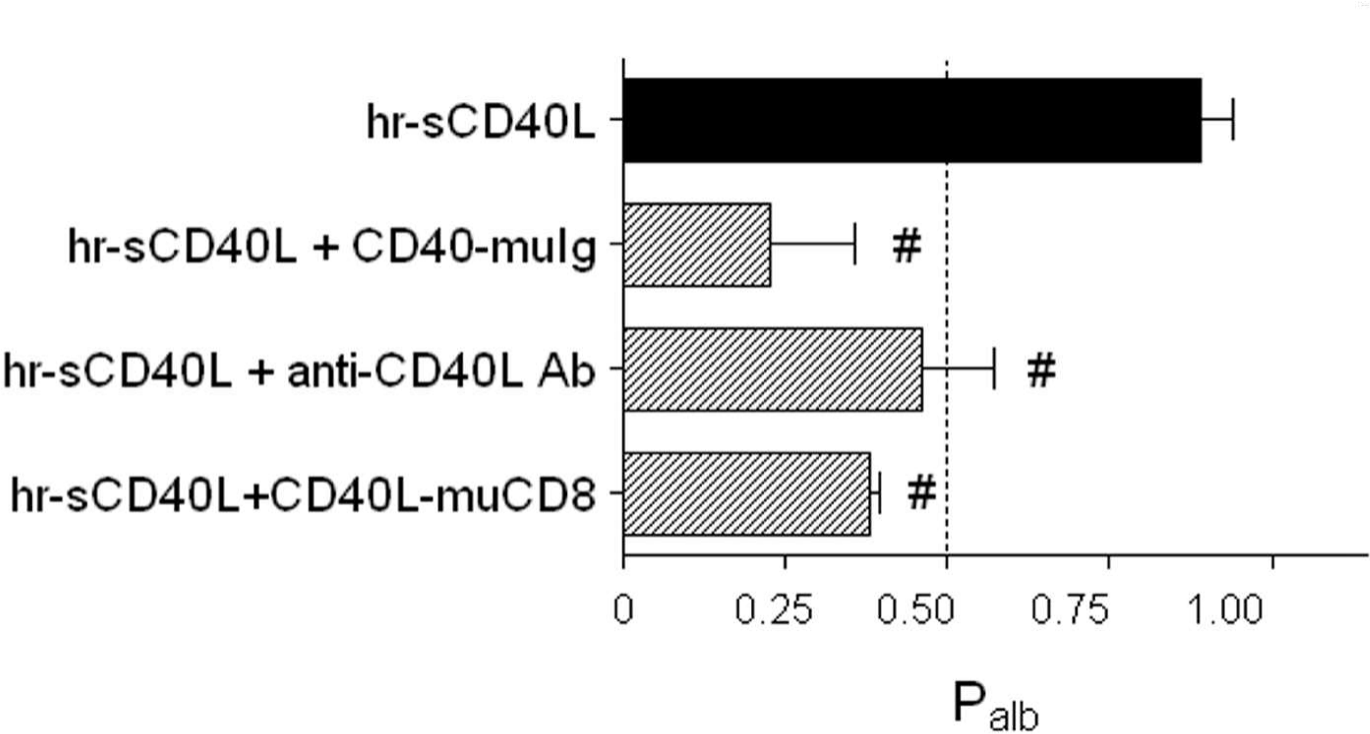
Permeability activity of albumin (P_alb_) induced by sCD40L in isolated rat glomeruli. P_alb_ was determined after the incubation of rat glomeruli for 30 min with hr-sCD40L (100 ng/ml + 1 μg/ml enhancer). A significant increase in glomerular permeability is expressed by values of P_alb_ greater than 0.5 (black bar). Using CD40-muIg fusion protein (20 ng/ml) and the neutralizing antibody against CD40L (5 μg/ml), hr-sCD40L was pre-treated for 10 minutes prior to glomerulus stimulation, whereas the CD40L-muCD8 fusion protein (50 ng/ml) was added to the glomeruli 30 minutes before adding hr-sCD40L (dashed bars) (see Methods for details). At least five animals were studied per each experimental group. *P < 0.05 and **P < 0.01 versus unstimulated controls; ^#^*P* < 0.01 versus rh-sCD40L.

The effect of sCD40L on glomerular permselectivity was evaluated by using an *ex-vivo* bioassay based on measuring the increase in albumin permeability (P_alb_) in isolated rat glomeruli [19, 33]. sCD40L induced a significant increase of P_alb_, expressed by values greater than 0.5 (Fig 3; black bar), which was blunted by pre-treating sCD40L with an inhibitory CD40-muIg fusion protein or a neutralizing antibody against CD40L, or by pre-incubating the glomeruli with a CD40L-muCD8 fusion protein (Fig 3, dashed bars).

### Effect of in vivo injection of sCD40L

**Fig 4:**
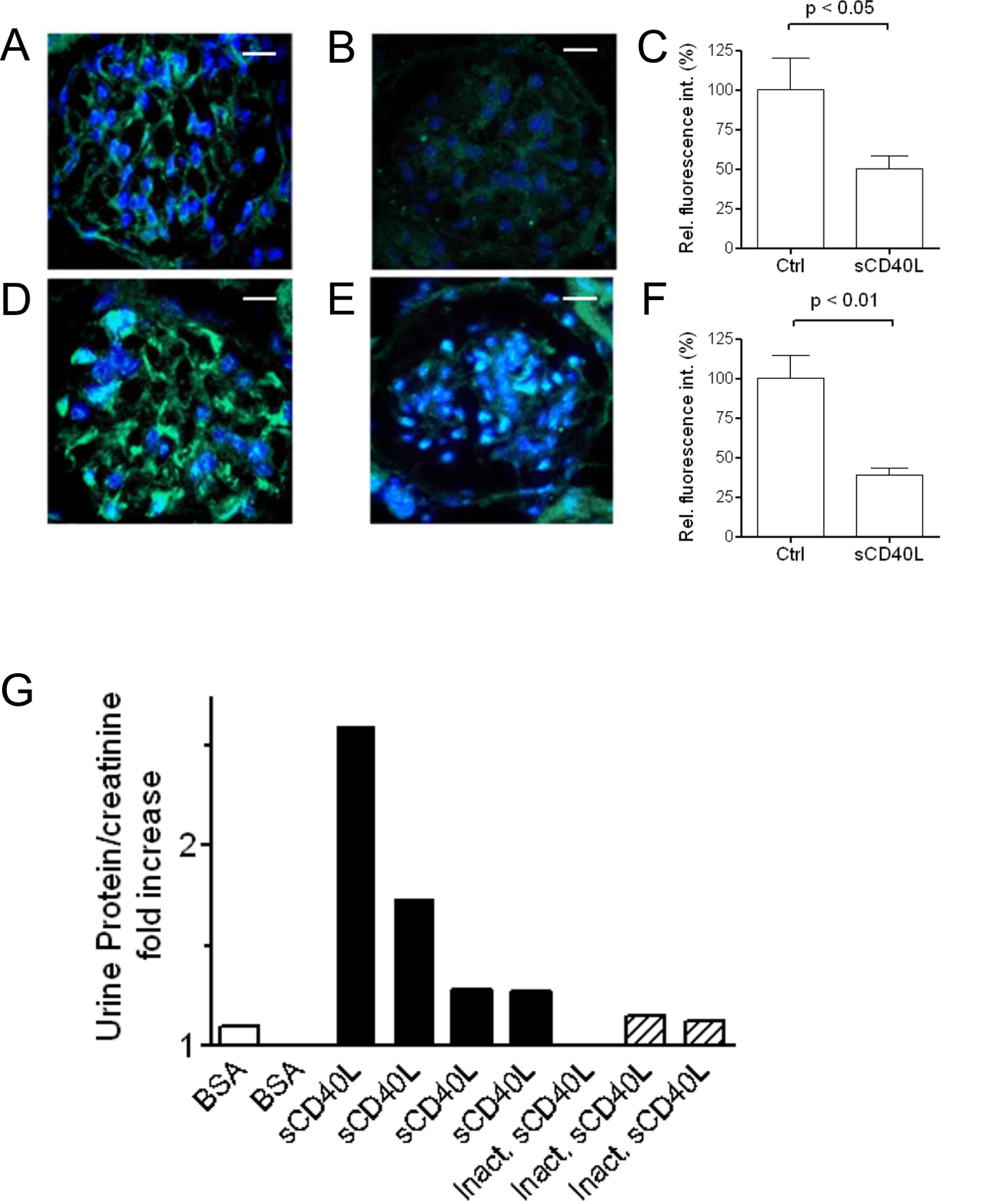
**(A-F) Effect of *in vivo* injection of sCD40L on the glomerular expression of nephrin and podocin.** (A-B) Micrographs representative of immunofluorescence staining for nephrin in glomeruli from female C57BL/6 mice injected *in vivo* with 200 ng sCD40L (C) or, as negative control, 200 ng heat-inactivated sCD40L (B) (x63). Bars = 10 μm. (C) Semiquantitative analysis of nephrin expression as detected by immunofluorescence staining in glomeruli from female C57BL/6 mice injected *in vivo* with sCD40L or, as negative control, heat-inactivated sCD40L (Ctrl). (D-E) Micrographs representative of immunofluorescence staining for podocin in glomeruli from female C57BL/6 mice injected *in vivo* with 200 ng sCD40L (F) or, as negative control, 200 ng heat-inactivated sCD40L (E) (x63). Bars = 10 μm. (F) Semiquantitative analysis of podocin expression as detected by immunofluorescence staining in glomeruli from female C57BL/6 mice injected *in vivo* with 200 ng sCD40L or, as negative control, 200 ng heat-inactivated sCD40L (Ctrl). Glomerular expression of nephrin and podocin was evaluated by indirect immunofluorescence by confocal microscopy on frozen kidney sections obtained twenty-four hours after injection, as detailed in the Methods section. Six animals were studied per experimental group. (G) Urine protein/creatinine ratio 24 hours after a single injection of 5% bovine serum albumin (BSA–white bars), sCD40L (black bars), or heat-inactivated sCD40L (dashed bars) in female C57BL/6 mice. Each bar is representative of a single experiment.

*In vivo i*.*v*. administration of sCD40L, but not of heat-inactivated sCD40L, induced a marked decrease in nephrin expression in mouse glomeruli at 24 hours from the injection (Fig 4, panels B and C). Analogously, the injection of sCD40L, but not of heat-inactivated sCD40L, through the tail vein provoked a profound reduction of the glomerular expression of podocin (Fig 4, panels E and F). However, *in vivo i*.*v*. injection of sCD40Ldid not induce a significant increase in urine protein/creatinine ratio (Fig 4G).

### Inhibition of CD40-CD40L interaction abrogates the effect of PF from patients with FSGS on nephrin expression in cultured GECS

**Fig 5:**
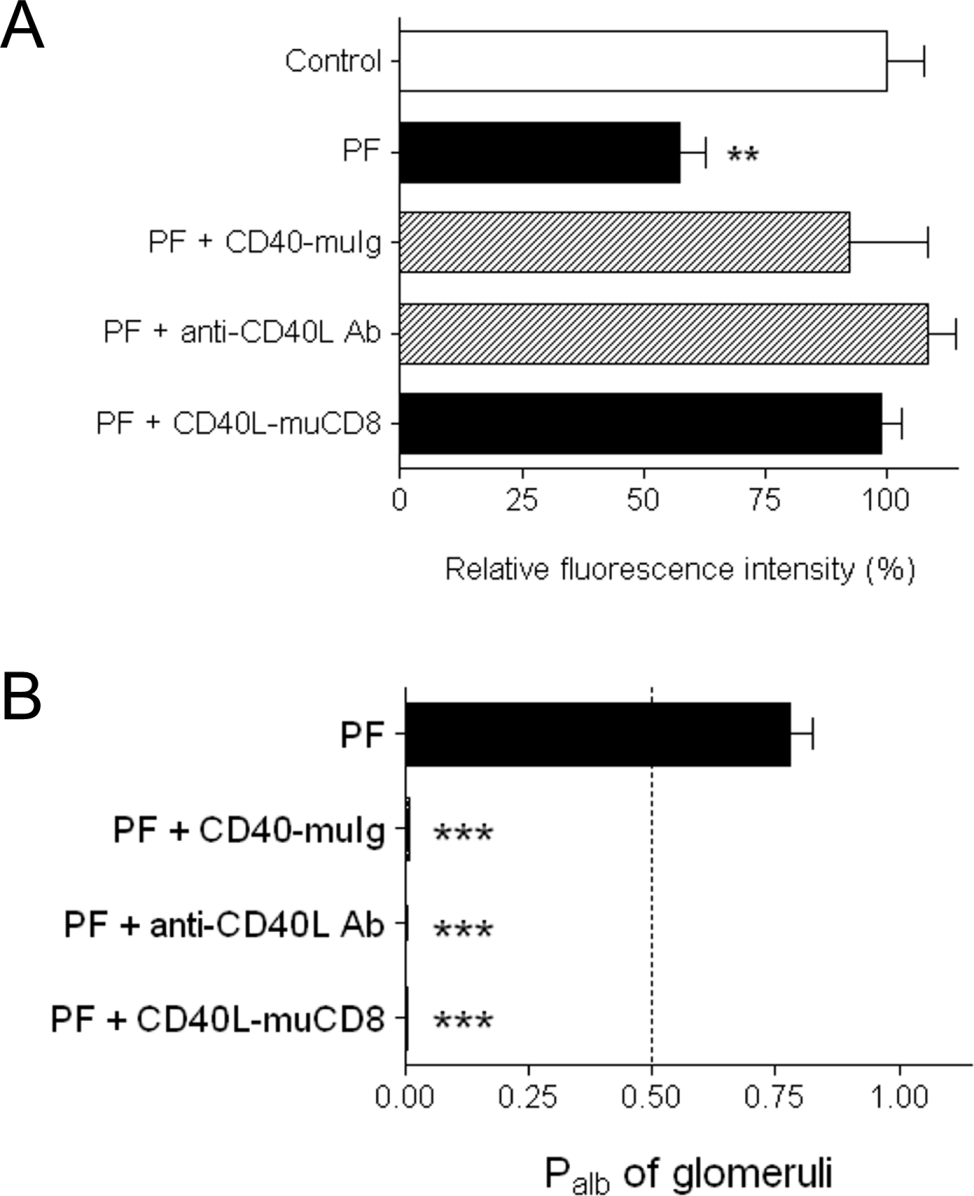
**Effect of the partially purified Permeability Factor (PF), prepared from plasmapheresis eluates from patients who presented post-transplant recurrence of FSGS, on nephrin expression in cultured GECs (A) and on Permeability activity of albumin (P**_**alb**_**) in isolated rat glomeruli (B).** (A) Semiquantitative analysis of nephrin expression as detected by immunofluorescence staining in GECs incubated with PF (500 ng/ml) for 30 min in the absence (black bar) or presence of different inhibitors of CD40-CD40L interaction. Using CD40-muIg fusion protein (20 ng/ml) and the neutralizing antibody against CD40L (5 μg/ml), hr-sCD40L was pre-treated for 10 minutes prior to GEC stimulation, whereas the CD40L-muCD8 fusion protein (10 μg/ml) was added to cultured GECs 30 minutes before adding hr-sCD40L (see Methods for details). **P < 0.01 versus unstimulated control and PF + inhibitors of CD40-CD40L interaction. Values are derived from 5 or more experiments for each experimental condition and expressed as percent variations from baseline value. (B) Permeability activity of albumin (P_alb_) induced by PF on isolated rat glomeruli: P_alb_ was determined after the incubation of rat glomeruli with PF (500 ng/ml), as detailed in the Methods section. A significant increase in glomerular permeability is expressed by values of P_alb_ greater than 0.5 (black bar). Using CD40-muIg fusion protein (20 ng/ml) and the neutralizing antibody against CD40L (5 μg/ml), hr-sCD40L was pre-treated for 10 minutes prior to glomerulus stimulation, whereas the CD40L-muCD8 fusion protein (10 μg/ml) was added to the glomeruli 10 minutes before adding PF. At least five animals were studied per each experimental group.

We then examined whether sCD40L could represent the so-called Permeability Factor (PF) previously described as crucial effector of proteinuria in primary FSGS and post-transplant recurrence of FSGS [20–23]. This putative molecule described in FSGS patients shares with sCD40L many structural and biological features, such as the production by activated T-lymphocytes, and its molecular weight [1–4]. Therefore, we have evaluated whether the effects of the partially purified PF in GECs could be prevented by inhibiting the biological activity of sCD40L. In this part of the study, we utilized a partially purified plasmatic fraction prepared from plasmapheresis eluates from 6 patients who presented post-transplant recurrence of FSGS (Table 1). The inhibition of CD40-CD40L interaction completely abrogated the effects of the purified PF on nephrin expression in GECs, as shown both by indirect immunofluorescence (Fig 5A) and Western blot (Fig 2F).

### Inhibition of CD40-CD40L interaction abrogates the effect of PF from patients with FSGS on glomerular permselectivity in isolated rat glomeruli

Analogously to what we observed in cultured GECs, the inhibition of CD40-CD40L interaction abrogated the increase of P_alb_ induced by purified PF in isolated rat glomeruli (Fig 5B).

### Effect of in vivo injection of PF from patients with FSGS on glomerular expression of nephrin

**Fig 6:**
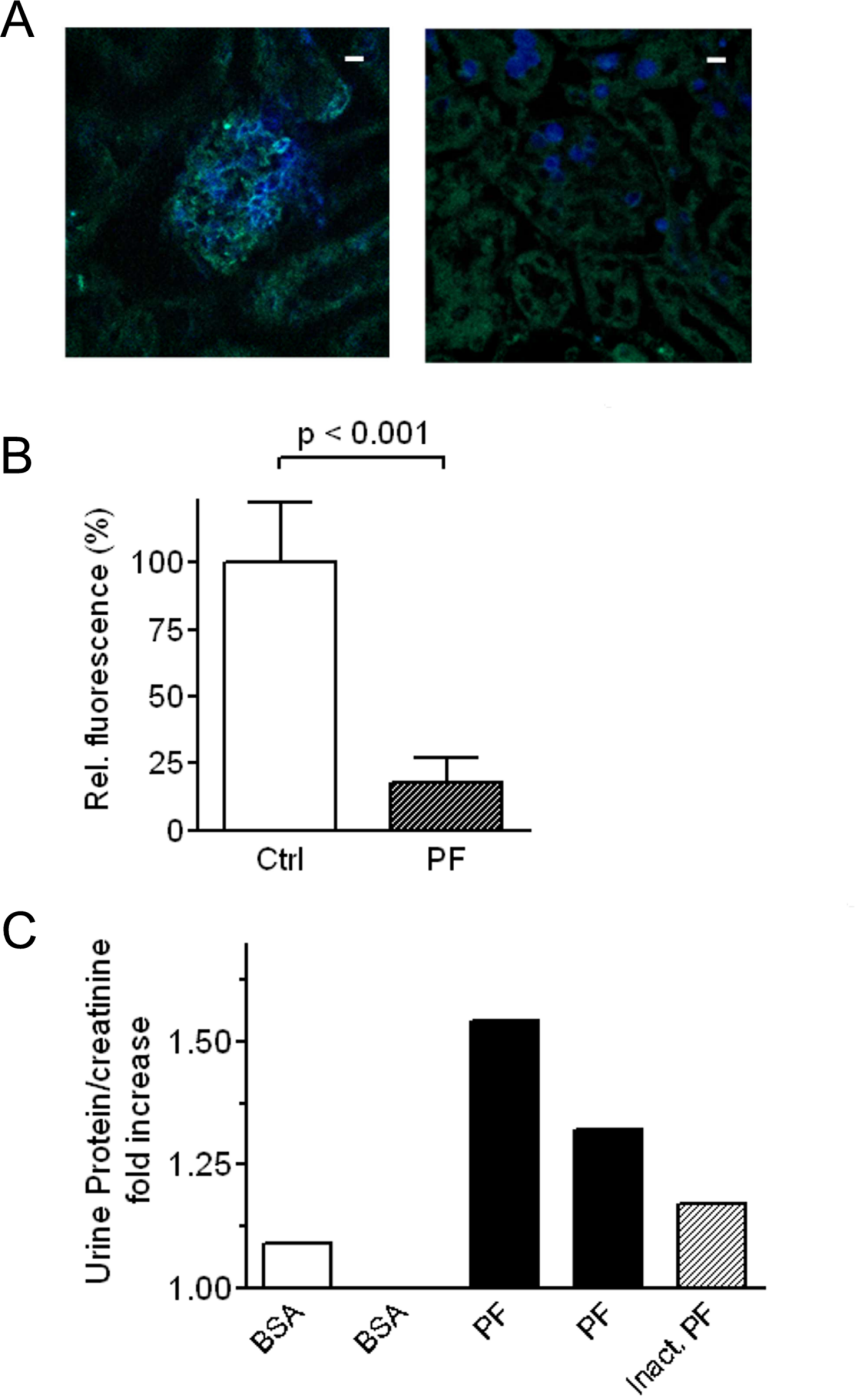
Effect of *in vivo* injection of PF on the glomerular expression of nephrin and urine protein/creatinine ratio. (A) Micrographs representative of immunofluorescence staining for nephrin in glomeruli from female SCID mice injected *in vivo* with PF (left image) or, as negative control, heat-inactivated PF (right image) (x63). Bars = 10 μm.(B) Semiquantitative analysis of nephrin expression as detected by immunofluorescence staining in glomeruli from female SCID mice injected *in vivo* with PF or, as negative control, heat-inactivated PF (Ctrl). At least five animals were studied per each experimental group. (C) Urine protein/creatinine ratio 24 hours after a single injection of PF (black bars) or heat-inactivated PF (dashed bar) in female SCID mice. Each bar is representative of a single experiment.

Analogously to what we observed with sCD40L, the *in vivo i*.*v*. administration of PF, but not of heat-inactivated PF, induced a marked decrease in nephrin expression in mouse glomeruli (Fig 6, panels A and B). However, i*n vivo* PF injection did not induce a significant increase in urine protein/creatinine ratio at 24 hours from the injection (Fig 6C).

### Detection of sCD40L in plasma fractions obtained by plasmapheresis eluates of FSGS patients

**Fig 7:**
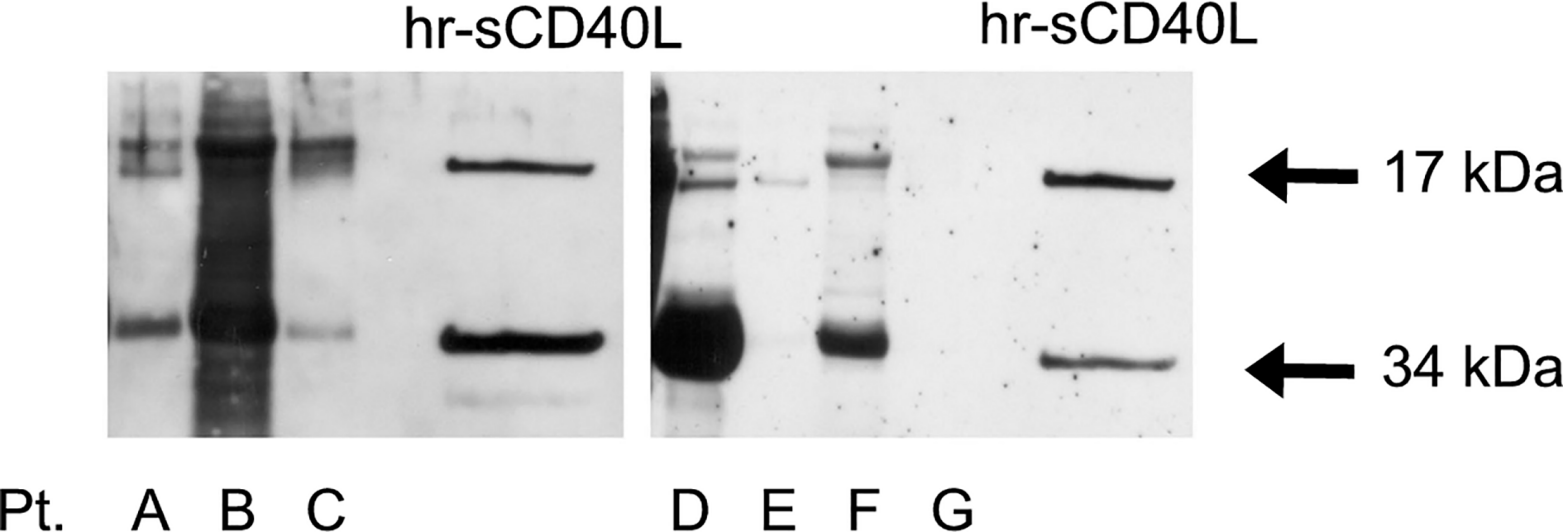
Detection of sCD40L in plasma fractions obtained by plasmapheresis eluates of FSGS patients by Western blotting. Western blot analysis of sCD40L in PF preparations obtained from plasmapheresis eluates of different FSGS patients with elevated P_alb_ (lanes A-F), and from a normal patient with negative P_alb_ (lane G). In the former case, PF was prepared utilizing plasma from FSGS patients undergoing plasmapheresis and separated on Protein A at different conditions; in the second case, normal plasma without P_alb_ activity was obtained as unbound to Protein A material. PF preparations (5μg) were immunoblotted with anti-sCD40L antibody, which identified two bands, at 17 and 34 kDa, corresponding to the monomeric and dimeric forms of the molecule, respectively. hr-sCD40L (500 ng) was used as positive control. Each lane corresponds to an individual patient.

Western blotting analysis of plasma fractions prepared from plasmapheresis eluates obtained from different patients with post-transplant recurrence of FSGS revealed the presence of two bands, at 17 and 34 kDa, identical to those of human recombinant sCD40L, which correspond to the monomeric and dimeric forms of sCD40L (Fig 7). The pre-adsorption of the antibody with human recombinant sCD40L completely prevented the appearance of the two bands, showing the specificity of the signal detected by Western blotting (data not shown).

### sCD40L is increased in serum of patients with FSGS

Serum sCD40L was measured in several cohorts of children with nephrotic syndrome (NS), subdivided according to the sensitivity to steroids, all with preserved renal function (eGFR >60 ml/min). Overall, 38 children were studied, 23 with steroid-dependence, 15 with steroid-resistance, who were treated with a multidrug approach and had a renal biopsy.

Children who developed NS under the 1^st^ year of age, defined as congenital nephrotic syndrome (cNS), underwent a bioptic approach, and were studied with molecular tests. ACTN4 and TRCP mutations were found in two cases.

All adults with NS had a renal biopsy at the onset of proteinuria, and those presenting FSGS were treated with steroids in association with calcineurin inhibitors. Forty adults had a histology diagnosis of iMN, and underwent a different therapeutic approach.

**Fig 8:**
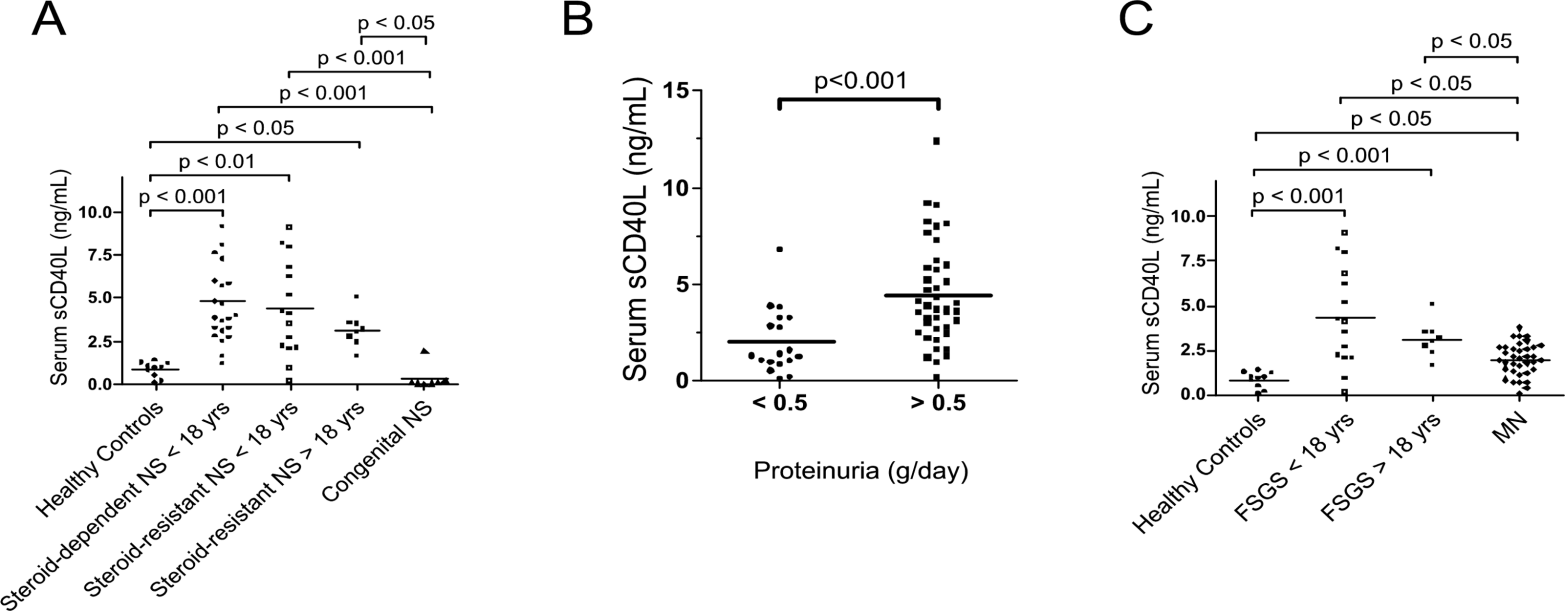
sCD40L concentrations in serum samples from children and adult patients with idiopathic nephrotic syndrome and from healthy subjects. (A) Serum sCD40L levels in healthy controls and in patients with idiopathic nephrotic syndrome (NS), subdivided according to clinical features (*i*.*e*. age at onset and response to steroids). Baseline clinical characteristic of the patients with idiopathic nephrotic syndrome are reported in Table 2. Children who presented proteinuria under the 1^st^ year of age underwent biopsy and had a diagnostic molecular approach for several genes implicated in NS; they were classified as congenital NS (cNS). (B) Serum sCD40L levels in healthy controls and in patients with steroid-resistant idiopathic nephrotic syndrome according to the levels of proteinuria (< or > 0.5 g/day). (C) Serum sCD40L levels in patients with nephrotic syndrome and a biopsy-proven diagnosis of FSGS or of iMN. Patients with FSGS were further subdivided according to their age in < or > 18 years. Patients with iMN were always older than 40 years.All patients had eGFR >60 ml/min at the time of serum sampling for sCD40L measurement.

Data on serum levels of sCD40L are given in Fig 8. In general, sCD40L levels were higher in patients with NS than in healthy subjects (Fig 8, panels A and C). Moreover, sCD40L was significantly higher in patients both with steroid-dependent and with steroid-resistant NS, at any age, than in healthy subjects (Fig 8A), and in patients with steroid-resistant NS who had proteinuria >0.5 than in those with proteinuria <0.5g/day (Fig 8B). However, we did neither found a direct correlation between sCD40L and proteinuria, nor between sCD40L and eGFR. On the contrary, the patients with congenital NS had very low sCD40L levels (Fig 8A). In addition, patients with biopsy-proven FSGS, at any age, had significantly higher sCD40L concentrations than healthy subjects (Fig 8C). Finally, sCD40L in patients with membranous nephropathy, although slightly, but still not significantly, higher than in the healthy subjects, resulted significantly lower than in patients with steroid-resistant NS (Fig 8C).

## Discussion

In this study, we describe a novel role for sCD40L as a modifier of glomerular permselectivity that acts directly on GECs. The expression of CD40 by GECs has been previously reported by several research groups [9, 14–17]. Delville M. and coll. [14] have shown CD40 expression in cultured human podocytes and on podocyte surface in kidney tissue of FSGS patients, but not of normal subjects [14]. Other studies have independently confirmed that podocytes express CD40 on their plasma membrane, both in human kidney tissue sections, by using immunohistochemistry and electron microscopy [15], and in rat [16] and mouse glomeruli [9]. Moreover, a recent paper by Kuo H.L. and coll. demonstrated that podocytes express CD40 and that its stimulation by CD40L up-regulates inflammatory mediators in the context of early diabetic nephropathy [17]. In our study, we were able to confirm, by different techniques, that human, mouse and rat cultured podocytes, as well as glomeruli of normal mice and rats express CD40. In addition, we have shown that CD40 stimulation by sCD40L induces several biological effects, suggesting a central role for CD40-CD40L dyad in regulating glomerular permselectivity. sCD40L induces, indeed, the redistribution and loss of nephrin, and the reorganization of cell cytoskeleton in cultured GECs (phenomena previously associated with the development of proteinuria [33, 35, 39]), alters the permselectivity of isolated rat glomeruli, and, when injected *i*.*v*. in mice, markedly reduces nephrin and podocin glomerular expression.

Based on these experimental results, we hypothesized that sCD40L could represent the so-called Permeability Factor (PF), which is considered a crucial effector of proteinuria in primary FSGS and post-transplant recurrence of FSGS, but, notwithstanding the research effort of several groups, had not been identified yet [20–29]. It has been previously shown that the putative PF may be bound to an immunoglobulin, if it is not an immunoglobulin itself [41], and has a molecular weight of 30–50 kDa [42]. In the last 15–20 years several molecules have been proposed as potential candidate circulating PF, either circulating or locally produced within the glomerulus, including cytokines and growth factors such as vascular permeability factor (VPF) [43], tumor necrosis factor (TNF)-α [44], transforming growth factor (TGF)-β [45], and hemopexin [46]. In the last years, soluble urokinase receptor (suPAR) has received extensive attention, since it increases in patients with recurrent FSGS and correlates with disease activity, although not all the molecular forms of suPAR were shown to be equally pathogenic for podocytes [47–50]. In addition, cardiotrophin-like cytokine (CLCF)-1, a member of IL-6 family of cytokines produced by T-cells, has been recently identified as a candidate cytokine based on its ability to mimic the P_alb_ activity of FSGS sera *in vitro* [51]. Finally, several autoantibodies, for instance directed against actin, adenosine triphosphate synthase, angiotensin II type 1 receptor, protein tyrosine phosphatase receptor type O (PTPro), and nephrin, some of them also showing the ability to increase P_alb_ [52], have also been recently implicated in FSGS pathogenesis [52–55]. Interestingly, autoantibodies against PTPro have been also identified in a recent study in sera of patients with recurrent FSGS [14].

Since the putative PF described in FSGS patients shares with sCD40L many structural and biological features, such as the production by activated T-lymphocytes, and its molecular weight [1–4], we hypothesized that the partially purified PF isolated from plasmapheresis eluates of FSGS patients could contain sCD40L. The results of the present study showed that blocking the CD40-CD40L dyad prevents the biological effects of PF both in GECs (nephrin loss and cytoskeleton reorganization) and in isolated glomeruli (P_alb_ increase). Further support to our hypothesis derives from the observation that the *in vivo i*.*v*. injection of PF, but not of heat-inactivated PF, induced a marked decrease in nephrin expression in mouse glomeruli, although not associated with increased proteinuria. Finally, we have directly shown by Western blotting the presence of sCD40L in plasma fractions prepared from plasmapheresis eluates obtained from different patients with post-tranplant recurrence of FSGS. Taken together, these results suggest that sCD40L may be identified as a soluble PF, whose existence in FSGS patients has been widely hypothesized, but never clearly documented. Our results are in line with and independently confirm those reported by Delville M. and coll. [14], who have shown that anti-CD40 antibodies purified from patients with recurrent FSGS are pathogenic in cultured podocytes, and enhance *in vivo* the proteinuric effect of suPAR injection [14]. Therefore, in addition to autoantibodies directed against CD40, also the presence of high circulating levels of sCD40L could contribute to the pathogenesis of FSGS engaging the GEC-expressed CD40 receptor.

We then measured the concentrations of sCD40L in pediatric and adult patients with NS, due to steroid-dependent and steroid resistant NS, or membranous nephropathy, and, as negative controls, in healthy subjects. sCD40L was significantly more elevated in pediatric and adult patients affected by both steroid-dependent and steroid-resistant NS than in healthy subjects. Patients with FSGS, at any age, had higher sCD40L than healthy subjects, while children with congenital NS had very low sCD40L levels. Finally, patients with iMN had sCD40L concentrations significantly lower than those measured in patients with steroid-resistant NS. These results, although preliminary, seems coherent with the hypothesized role of sCD40L as permeability factor acting specifically in glomerular diseases affecting podocyte function, such as FSGS.

A tendency versus lower concentrations of sCD40L in older patients than in children could be also noted. We have no clear explanation for this result that could be, at least partially, due to a generically less pronounced inflammatory reactivity in older people.

Since we could not find a correlation of sCD40L levels with eGFR, it seems reasonable to exclude that increased serum sCD40L would be secondary to reduced filtration, as hypothesized for other putative permeability factors, such as suPAR [56]. However, larger studies would be needed to definitely address this issue.

Several limits should be taken into account in interpreting our results on circulating sCD40L levels. First, we did not strictly standardize, mainly due to the difficulties secondary to the low incidence and clinical heterogeneity of the disease, the exact timing of serum sampling for the measurement of sCD40L levels. In addition, neither we systematically studied sCD40L before and after transplantation, nor before and after plasmapheresis therapy. Finally, the exclusion of patients with severely damaged/loss of renal function does not allow us to definitely rule out the hypothesis that circulating sCD40L may also increase, at least partially, as a consequence of reduced clearance of sCD40L due to a reduction of the filtration rate. For all these reasons, our clinical results must be considered preliminary and need to be confirmed in larger studies. Interestingly though, a recent study has shown that plasma levels of circulating CD40 are negatively associated, whereas sCD40L levels are directly associated with declines in eGFR in an all-cause chronic kidney disease cohort [57], suggesting that the increase in sCD40L levels may precede the loss of renal function.

High levels of sCD40L have been described in several inflammatory diseases [1–4, 58–60]and cancer [61–63]. Yet, these conditions are not automatically associated with proteinuria, thus questioning the possible causative role of elevated sCD40L in altering glomerular permselectivity. Interesting observations potentially useful to explain these apparently contradictory data derive from the study by Delville and coll. [14] Anti-CD40 antibodies prepared from patients with recurrent FSGS, indeed, do not recognize human CD40, yet they are able to produce podocyte damage *in vitro* and *in vivo* [14]. This effect may be related to the unmasking of cryptic podocyte epitopes caused by other inflammatory stimuli simultaneously acting on this cell-type, a condition not present in other diseases not affecting the kidney. The same can be hypothesized for sCD40L, which could access to its receptor CD40 and induce proteinuria only acting in an additive or synergistic manner with other mediators. Furthermore, the same authors suggested that a perturbation in the conformation of the CD40 protein may be needed in order to alter its immunogenicity and cause the production of pathogenic anti-CD40 autoantibodies [14]. This phenomenon could also be needed for sCD40L binding and the full expression of its pathogenic effect.

The *in vivo* injection of sCD40L and PF, although able to induce a reduction in nephrin expression, did not induce a significant increase in proteinuria, although, in 2 out of the 4 animals injected with sCD40L and in both mice injected with PF, urine protein/creatinine ratio was slightly increased. This result could be ascribed to different factors. First, recombinant sCD40L and sCD40L contained in the partially purified fractions of plasmapheresis eluates could not be able to reach, *in vivo*, the concentrations needed to effectively stimulate CD40. Moreover, the lack of a concomitant inflammatory “milieu” in normal animals could prevent sCD40L and PF from inducing a significant proteinuric effect *in vivo*. Inflammatory signals enhance, indeed, CD40 expression [1–4], which could not result high enough in normal mice. Furthermore, the presence of other inflammatory mediators may be required for sCD40L to trigger proteinuria, as already shown for anti-CD40 antibodies purified from patients with recurrent FSGS, whose administration in mice was not sufficient *per se* to cause robust albuminuria, but enhanced the proteinuria induced by suPAR [14].

Finally, we must acknowledge that we only studied the effect of sCD40L and PF infusion at 24 hours, a time when histologic damage could not be expected to develop. Although we were able to show that acute injection of sCD40L and PF reduced nephrin expression and increased urine protein/creatinine ration in some animals, we do not know whether repeated or continuous administration of sCD40L or PF would be able to induce an FSGS-like glomerulosclerosis over longer time scales.

Taken together, our results suggest that sCD40L could be indicated as an additional putative PF involved in the pathogenesis of primary and/or recurrent FSGS. Moreover, they seem coherent with the hypothesis that more than one mediator, either circulating or locally produced within the glomerulus, could participate in this process, or even an entire cytokine “milieu” need to be present, as already pointed-out by other authors [14, 44]. This condition could eventually make even more difficult to find a strong correlation between the concentration of a putative PF and disease severity or proteinuria.

In conclusion, the results of our study suggest that sCD40L may directly affect podocyte function interacting with its receptor CD40, and act as a circulating permeability factor that can participate, presumably in collaboration with other mediators, in the pathogenesis of FSGS. Further studies are needed in order to elucidate the mechanisms of CD40 activation in spontaneous human glomerular pathology, and to develop possible new therapeutic approaches to block the CD40-CD40L axis with the aim to inhibit progression and/or recurrence of FSGS.
